# An anthropological history of Nepal’s Female Community Health Volunteer program: gender, policy, and social change

**DOI:** 10.1186/s12939-024-02177-5

**Published:** 2024-04-13

**Authors:** Roosa Sofia Tikkanen, Svea Closser, Justine Prince, Priyankar Chand, Judith Justice

**Affiliations:** 1 Institute of Sociology and Political Science, Faculty of Social and Educational Sciences, Norwegian University of Science and Technology, Edvard Bulls veg 1, 7491 Trondheim, Norway; 2 Department of International Health, Johns Hopkins Bloomberg School of Public Health, 615 N. Wolfe Street, Baltimore, Maryland 21205 USA; 3 Zanvyl Krieger School of Arts & Sciences, Johns Hopkins University, 3400 N. Charles Street, Baltimore, Maryland 21218 USA; 4 Institute for Health & Aging, School of Nursing, University of California at San Francisco, 490 Illinois Street, San Francisco, CA 94143 USA

**Keywords:** Community health workers, Nepal, Gender, Volunteerism, Labor movements, Health workforce policy, Primary Health Care, Anthropology

## Abstract

**Background:**

Community health workers (CHWs) are central to Primary Health Care globally. Amidst the current flourishing of work on CHWs, there often is a lack of reference to history—even in studies of programs that have been around for decades. This study examines the 35-year trajectory of Nepal’s Female Community Health Volunteers (FCHVs).

**Methods:**

We conducted a content analysis of an archive of primary and secondary research materials, grey literature and government reports collected during 1977-2019 across several regions in Nepal. Documents were coded in MAXQDA using principles of inductive coding. As questions arose from the materials, data were triangulated with published sources.

**Results:**

Looking across four decades of the program’s history illuminates that issues of gender, workload, and pay—hotly debated in the CHW literature now—have been topics of discussion for observers and FCHVs alike since the inception of the program. Following experiments with predominantly male community volunteers during the 1970s, Nepal scaled up the all-female FCHV program in the late 1980s and early 1990s, in part because of programmatic goals focused on maternal and child health. FCHVs gained legitimacy as health workers in part through participation in donor-funded vertical campaigns. FCHVs received a stable yet modest regular stipend during the early years, but since it was stopped in the 1990s, incentives have been a mix of activity-based payments and in-kind support. With increasing outmigration of men from villages and growing work responsibilities for women, the opportunity cost of health volunteering increased. FCHVs started voicing their dissatisfaction with remuneration, which gave rise to labor movements starting in the 2010s. Government officials have not comprehensively responded to demands by FCHVs for decent work, instead questioning the relevance of FCHVs in a modern, medicalized Nepali health system.

**Conclusions:**

Across public health, an awareness of history is useful in understanding the present and avoiding past mistakes. These histories are often not well-archived, and risk getting lost. Lessons from the history of Nepal’s FCHV program have much to offer present-day debates around CHW policies, particularly around gender, workload and payment.

**Supplementary Information:**

The online version contains supplementary material available at 10.1186/s12939-024-02177-5.

## Introduction

Community health workers (CHWs) are central to programs addressing health inequalities, as they are often tasked with serving underserved populations and are embedded in their communities. However, systematic literature reviews have found that the equity-promoting effects of CHW programs are often uneven [1–4]. This has been attributed to CHWs lacking adequate health systems supports, which may make it difficult for CHWs to meet programmatic goals [1–3, 5]. The most recent of these systematic reviews highlighted that working conditions are central to CHW empowerment [3].

While topics of CHW working conditions, such as incentives, support and workload, have been topics of conversation around CHW programs since before the Alma-Ata declaration on Primary Care, much of this literature ignores the historical legacy of these programs – even in studies of CHW programs that have been around for decades.

In part, the lack of historical material in the literature may be a result of how difficult these histories are to access. The records of CHW programs before 2000 often only exist on paper and are not found online. Much of the content also exists outside of the peer-reviewed literature. In addition, early documents may have not been systematically archived in many countries. Thus, these histories risk being lost.

The few existing historical accounts of CHW programs offer helpful distinctions between programmatic issues that are temporally bound, versus those that have gone unresolved since their beginning, with the latter having the potential to inform current-day policy debates [6]. A historical view also allows for critical re-examination of mainstream discourses around a given topic [7].

This paper is an account of the 35-year history of Nepal’s most prominent CHW cadre, the Female Community Health Volunteers (FCHVs). This cadre has persisted as a stable government-engaged workforce through several cycles of political turmoil. The program has also been hailed as one of the most ‘successful’ CHW models brought to scale [8].

There is substantial contemporary peer-reviewed literature on FCHVs [9–15]. The majority of FCHV literature however comprises cross-sectional studies, and the few historical accounts that exist pertain to vertical, disease-specific programs [16–19].

CHWs globally are predominantly female [20–22]. Although gender roles are highly culturally-specific and time-bound, a systematic review identified several gendered issues that shape the experiences of CHWs such as safety and mobility, family and intrahousehold dynamics, and health systems hierarchies and power dynamics, including career progression and remuneration [23]. Although the gendered aspects of CHW work and information on labor movements is documented in the literature for other CHW cadres in South Asia [24–28], these dynamics remain largely unexplored in the academic literature on FCHVs, apart from one analysis [29].

Our analysis draws on the personal archives of Judith Justice, an anthropologist with a long connection to Nepal. Justice conducted several health systems research projects in Nepal, and her archive includes materials collected across five decades. Here, we review this material to present an anthropological history of the FCHV program, through the lens of Justice’s work. Our analysis runs from 1977 to 2019, covering a time period during which Nepal has experienced many transitions, epidemiologically, demographically, economically and culturally. Our goal is not to provide an update on the current status of FCHVs, but to provide insights on the historical context of the program.

During the 1970s, Nepal was one of the poorest countries in the world, at least if measured in GDP per capita terms [30]. Over the next decades, Nepal saw vast reductions in poverty and illiteracy, fertility, maternal and infant mortality [31–33]. During these decades, the country’s population size nearly tripled and life expectancy increased by about 20 years [32]. This period has also seen vast changes in the governance landscape, including the introduction of democracy following a People’s Movement (*jana andolan*, 1990), a violent decade-long civil war (1996-2006), three constitutions, the fall of monarchy (2008), and a shift to federalization with the 2015 constitution.

Our analysis contextualizes our findings within these larger sociopolitical shifts. In exploring the material, we explicitly adopt a gender lens, outlining how changes in women’s roles in the home and in the labor market have impacted FCHVs.

## Methods

Our analysis draws primarily on materials compiled by Judith Justice over the course of her career working in Nepal. This collection includes digital and paper-based materials from health research projects conducted in Nepal during 1977 - 2019.

A large proportion of our materials belonged to two major studies, both funded by the U.S. Agency for International Development (USAID) through Jhpiego and the Population Council. Both resulted in reports that are to the best of our knowledge not publicly available online; neither generated any peer-reviewed articles at the time.

The first study was conducted in 1992 by the Nepali non-profit research organization New ERA [34]. We refer to this as ‘the 1992 study’. Dr. Justice served as an external consultant for this ethnographic study which included semi-structured interviews, focus group discussions and participant observation conducted in Chitwan, Dhading, and Sarlahi districts, selected to represent a range of geographies across Hill, Terai (lowlands), and Inner Terai areas. Study participants included district health leaders; health post staff and managers; community members; and FCHVs.

The second study, on which Dr. Justice was also an external advisor, was conducted in 2001 by Jhpiego [35]. We refer to this as ‘the 2001 study’. Following a literature review, interviews were conducted in Dhading, Doti, Kanchanpur, Makwanpur, and Okhaldhunga districts with FCHVs, other community-based health volunteers and workers, community members, FCHV family members, private health practitioners, local government officials, District level officials, facility-based health workers, donor agencies, INGOs and NGOs. The fieldwork for this study was significantly impacted by the 1996-2006 civil war (Maoist Insurgency) and the 2001 Nepalese Royal Massacre in which nine members of the monarchy were killed.

A third study from which we drew upon was a smaller study on polio eradication in Rautahat district conducted by Dr. Justice and funded by the Bill and Melinda Gates Foundation; some results from this study have been previously published in peer-reviewed literature [36].

Our analysis aimed to review materials from this archive pertaining specifically to the FCHV program. Among the 172 documents in the archive that pertained to the FCHV program were: final and draft versions of reports written by non-governmental organizations (NGOs), international NGOs (INGOs) and bilateral agencies; government agencies; interview notes; interview transcripts; field notes; newspaper articles; training manuals; presentations; email printouts; faxes; books; and peer-reviewed research articles. Once scanned and digitized, we classified each document in terms of title, year, location of study, and our assessment of the quality and usefulness (richness) of the material for understanding the history of Nepal’s FCHV program. We used MAXQDA to code the most useful 111 documents by decade, as shown in Table 1.

**Table 1:** Overview of type and year of coded documents

	1980-1989	1990-1999	2000-2009	2010-2019	Undated	**Total**
**Primary data materials:** interview transcripts and notes; emails; faxes; field notes.	0	25	36	11	0	**72**
**Grey literature:** published and draft reports by NGOs, INGOs, the Ministry of Health and Population and regional governments; case studies: conference proceedings.	1	17	6	5	0	**29**
**Other:** training materials; newspaper articles; fieldwork materials (e.g. itineraries); peer-reviewed articles; books; written feedback received on reports, presentations and drafts.	1 ^a^	4	3	1	1	**10**
**Total**	**2**	**46**	**45**	**17**	**1**	**111**

^a^Note: Refers to a book published in 1989 based on fieldwork conducted in Nepal during the late 1970s

Analysis followed principles of inductive coding, investigating the material for emerging themes and subsequently coding these into higher-order categories (see Additional file 1). Although this study focused on the data in Dr. Justice’s archives and was not intended to be a comprehensive literature review, we did supplement our data using published sources (grey and peer-reviewed literature) when questions arose from our archival materials during thematic analysis. We structure our results chronologically and contextualize findings within the country’s sociopolitical trajectory.

The authors are mostly female and have US, European and Nepali backgrounds. While several have extensive fieldwork experience in Nepal, only one of the authors is a native Nepali speaker.

This research was reviewed and approved by the Johns Hopkins Bloomberg School of Public Health Institutional Review Board (#00019573).

### Findings

**Fig. 1:**
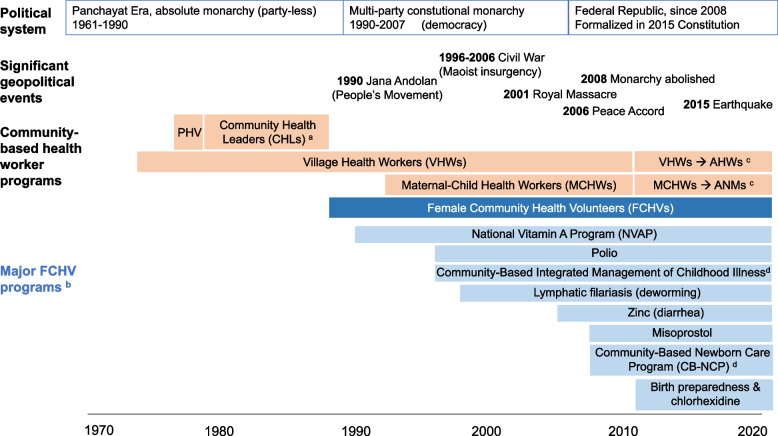
Timeline of Community Health Worker cadres in Nepal, and major Female Community Health Volunteer programs. Abbreviations: AHWs, Auxiliary health workers. ANMs, Auxiliary nurse midwives. PHV, Panchayat Health Volunteers. Footnotes: ^a^Also called Village Health Leaders (VHLs). ^b^ These include programs that were implemented nationwide or in a large number of districts. While other major FCHV health programs exist, they did not arise as strong themes in our materials and are hence not reviewed here. ^c^ 2011: VHWs with several years of experience ‘upgraded’ to facility-based AHWs, experienced MCWHs upgraded to facility-based ANMs. ^d^ The CB-IMCI program was integrated with the CB-NCP program into the Community-Based Integrated Management of Newborn and Childhood Illnesses (CB‐IMNCI) program in 2015. Sources: [19, 32, 37–40]

Nepal has a long history of state-sponsored CHWs (Fig. 1).

### Early experiments in community health workers

During the 1950s and 1960s, Nepal introduced several CHW cadres with specific health tasks, such as smallpox vaccinators (1967), family planning workers/health aides (1968), [31] and malaria home visitors (1958). These cadres were supported by international donors and multilateral agencies including USAID, WHO, UNICEF and UNFPA [41, 42]. During the 1970s, these ‘vertical’ cadres were rolled into one comprehensive cadre following successful pilot testing of ‘multipurpose’ health workers under the Integrated Basic Health Services project [43, 44]. This led to the Integrated Community Health Program which introduced a salaried, full-time cadre of Village Health Workers (VHWs) across the country during the late 1970s. VHWs were to link communities to Health Posts, the lowest-level primary health care center in rural areas, via home visits. They were hired as full-time, salaried government workers. Most were men, and many were from outside their villages [45].

During the late 1970s, another cadre, Panchayat Health Volunteers (PHVs), were introduced in a handful of districts. Two PHVs were nominated per *panchayat* (village-level elected body). While *panchayats* were encouraged to nominate women, in practice most were men. The PHV program was short-lived. Conflicts arose over the political nature of selection processes, and two volunteers proved insufficient to serve the population of an entire *panchayat*.

Following the 1978 Alma Ata conference, amid global enthusiasm for community participation in Primary Health Care, Nepal introduced a new cadre of unpaid CHWs, Village Health Leaders (VHL), also known as Community Health Leaders (CHLs) or Community Health Volunteers, (CHVs) [18]. They assisted VHWs in providing primary health care, first aid and family planning services [34]. Unlike VHWs, CHLs were to be selected by health committees in their local communities. Drawing lessons from the under-staffing of the PHV program, nine CHLs per *panchayat* were to be nominated.

The gender of CHLs was debated in the design phase of the program. A bilateral agency representative who worked on the design of the program commented several decades later:

*Given the nature of the work, I recommended that the CHLs all be women. Most of the rest of the training unit thought that this was a good idea, but that it wouldn’t work because they could never – or rarely – get a woman to do it. They appeased me by including in the selection guidelines that a CHL should preferably be a woman, but if none were available, a man would be OK. The result, of course, was that about 95% or more of the first people recruited were men.*

Most of the approximately 5,000 CHLs recruited were men, with the exception of Baitadi district, where an experimental program required all-female CHLs, and Jumla district where another experimental project hired one-third women [46].

Although CHLs were envisioned as a locally sourced workforce that would strengthen the work of VHWs, [47] they faced cultural and programmatic challenges. Recruitment was done quickly, and although the program design was to have local health committees select CHLs, in reality, it was often the case that these community committees were never formed [45]. Training was also sometimes not adequately tailored to their abilities, and supervision from VHWs was also frequently inadequate [45, 48]. A report prepared for the World Bank cited these issues as major factors in CHL inactivity and high rates of dropout [45]. In addition, the report noted, although it had been hoped that the volunteer nature of the position would contribute to higher credibility of the CHL in the eyes of the community, the absence of incentives "was thought to be another constraint with the CHLs programme" [45].

There were also other challenges. The CHL’s job description involved a long list of duties—some that demanded skills beyond the level of VHWs—all to be conducted without pay within just 6 hours of work per week. And, cultural norms prohibited women from accepting treatment from male workers and discussing family planning with them [34, 49].

The CHL program’s perceived limited effectiveness led to its discontinuation in 1987 [34], although the final 2001 study report notes that “it was not possible to locate a formal evaluation of this program” [35]. Based on her work in the 1970s, Dr. Justice noted that the CHL program was an example of “a chain of events extending from policies promoted in international health agencies to the pressures on planners in Kathmandu and ultimately to unrealistic programs installed at the village level” [48].

### An all-female volunteer program

An evaluation of the experimental program in Baitadi district, where CHLs were all female, found that women were “better at relaying maternal and child health, and family planning messages than their male counterparts in other districts” [18]. This ultimately led to a push for an all-female program. In 1988, the Ministry of Health and Population (MOHP) introduced the Female Community Health Volunteer (FCHV) program to replace the CHLs. At the time, Nepal was one of only two countries in the world where life expectancy for women was below that of men [32, 35]. A government workplan from 1990 explained that the choice of female workers was aimed at addressing infant death and adverse pregnancy outcomes [50]. FCHVs were tasked with addressing these issues through providing education, immunization, family planning, and hygiene, in addition to providing basic first aid services and referring to higher levels where needed [51].

FCHVs were to be selected by Mothers' Groups, composed of local women identified by the VHW and Nepal Women’s Organizations Primary Committee (NWOPC), a political wing of the then-ruling Panchayat regime. Early government workplans mandated that priority in selecting FCHVs should be given to members of NWOPC, “provided she is married and willing to work as a volunteer” [51], even if these women did not fit the formal selection criteria.[34] Failing that, the government plans specified, a woman should be found who was local, had been married, was at least 25 years old, was motivated to work as a volunteer, had experience in health (perhaps as a trained birth attendant), and who was ideally literate.

FCHVs were to be Secretaries of the Mothers' Group, which should be convened by the FCHV monthly to discuss local health problems and support adoption of what the government saw as good practices. The Mothers' Group was envisioned as the mechanism through which the FCHV became accountable to their local people [34].

Recruitment was initially challenging. Fieldnotes from the 1992 study noted that in several districts, Mothers’ Groups came into existence only after the FCHV had been selected.

A representative of an INGO noted in a 2017 interview that “when the program started, nobody wanted to be an FCHV… It was low status – a nurse was a stigmatized position.” Training opportunities and an honorarium of 100 Nepali Rupees (NPR) a month were used to persuade women to serve as an FCHV;[34] this honorarium represented 7% of the government’s health budget [52].

FCHV selection was impacted by caste and class dynamics. In the 1990s, up to half of FCHVs in a given district were illiterate [53]. Literacy classes were provided by USAID during the early years of the program [54] or in some cases by international NGOs [55]*.* The 1992 study noted that although practices varied, in many cases FCHVs were from higher castes and were less likely to serve poorer groups. They also noted that trainings were provided in Nepali language, which FCHVs from some ethnic groups struggled to understand [34].

Where women could not be motivated to serve, pressure could be applied by health post staff, local political leaders and other local elites. An international NGO representative noted in 2017 that “the program started ‘forcefully’ so the FCHV was often the wife of a teacher.”

### Searching for identity and community legitimacy during political transition

In 1990, the People’s Movement (Jana Andolan) ended single-party monarchy rule and ushered in a new era of parliamentary democracy. By this time, 14,000 FCHVs had been deployed across the country [45].

The 1992 New ERA study noted that during the early years of the program (1988-1990), the FCHV program functioned relatively well, but that the change in government caused challenges.

Many FCHVs were NWOPC members, and thereby associated with the previous government by community members [34]. The end of the Panchayat rule meant the end of these organizations, which necessitated a change to FCHV selection criteria. Some selection criteria were relaxed, including adjusting the lower age limit to 20 years, and allowing unmarried women who otherwise met selection criteria to be eligible [50].

The 1992 study report noted that:

*Most CHVs have remained politically neutral and continued to serve the community. However, many communities have become more fragmented with polarized political groups, thus making it difficult for CHVs to serve people in all political parties* [34]*.*

These political processes, combined with the rise of party politics, made the FCHV a more political position in some cases. Handwritten notes taken during fieldwork for the 1992 study noted that respondents felt that “where service and politics are not mixed, [FCHVs] are successful.” The draft report of this study noted that some community members refused to take medicines from FCHVs representing opposite political parties, and harbored “doubts that the [FCHV] may be giving poison instead of medicine.” These sections were excised from the final report, which simply noted that “since 1990 and the political changes people had stopped going to CHVs for service because of political views”[34]. The report also commented, however, that the FCHV program “proved itself viable in spite of the change in political and government system.”

### The end of pay

At the start of the program, FCHVs were paid 100 NPR per month as an honorarium. The 1992 study described the value of the stipend:

*Although by Kathmandu standards a hundred rupees is not much, by village standards it represents significant purchasing power…. Particularly in households which have no source of cash income, the honorarium added to the CHVs credibility and status* [34].

But, in June 1990, just two years since the inception of the program and just a few months following *jana andolan*, the honorarium was phased out. The Ministry of Health said providing the honorarium was not possible within their budget [34]. A government workplan from 1990 [50] noted that "other means" including education were to be investigated to encourage FCHVs’ continued participation.

This had several negative consequences from the point of view of FCHVs. The 100 NPR allowance, while certainly not enough to remove FCHVs from poverty, had allowed some small measure of financial security, including a buffer against family criticism for working outside the home. Handwritten fieldnotes from the 1992 study note that after the honorarium ended, “most daughters in law [were] prohibited to work.” The final study report elaborated:

*Since the cessation of honorarium, many CHVs in all districts say they do not have the same freedom in the family as earlier. Husbands, mothers and fathers-in-law are not happy about CHVs spending time on the program without compensation, at the cost of their domestic responsibilities* [34].

Beyond family percussions, FCHVs felt demoralized by the end of pay. The 1992 final study report noted that “FCHVs were humiliated once it was stopped and with its cessation, there was the perceived withdrawal of Government appreciation” [34].

FCHVs were not alone in their frustration over the end of pay, with government health staff, predominantly male, expressing that they “felt guilty asking CHVs to continue performing their tasks when they are the only member of the health team to be unpaid although the expected workload is as great as paid workers such as the VHW” [34].

Complicating community dynamics further for FCHVs was the fact that community members thought FCHVs were full-time, paid workers. Radio messaging was eventually used in an attempt to clarify the withdrawal of remuneration and the scope of services provided by FCHVs [34].

### At the intersection of community and government health systems

During the early 1990s, the FCHV program was rolled out across the country, with national coverage by 1995. Community members especially valued the FCHVs’ provision of direct services, including the distribution of contraceptives, oral rehydration solution and immunizations [34]. FCHVs’ provision of curative care, in particular, built their credibility; they were most known for these tasks [56]. FCHVs were increasingly treated with respect and sometimes addressed as *‘doctorni’* (female doctor) or *masternididi* (female teacher) [34].

But FCHVs also conversely struggled with legitimacy as health workers, as well as with filling their envisioned role as a bridge between government health facilities and the community. This was related to the health system itself being in a nascent phase, struggling with issues of supplies and logistics. The 1992 final study report notes that once villagers reached the health posts, trained health workers were often absent, and did not treat them with respect [48]. These factors in turn discredited FCHVs and weakened their prestige [34]. A draft report of the 1992 study explained:

*The government is trying to develop people’s habit to visit the health posts for various disease through motivation and referral by the CHVs and to develop their faith on the government’s health system... but in the meantime have not been able to provide the health posts with required tools and medicines. As a result, rural people often get disappointed with the poor service provided to them after a long journey.*

This passage was excised from the final study report.

### Vitamin A and polio programs heightened FCHV visibility

By 1993, 27,000 FCHVs had been trained, covering 58 of Nepal’s then 75 districts [34]. FCHVs’ legitimacy began to shift in the early 1990s, which saw the start of FCHVs’ involvement in high-profile vertical programs.

The National Vitamin A Program (NVAP) was piloted in the early 1990s and reached all districts by 2002. It included twice-yearly administration of high-dose vitamin A capsules to children under the age of five, along with nutritional education campaigns. In 1997, Oral Polio Vaccine campaigns were integrated into the NVAP, to be administered by FCHVs during National Immunization Days [16].Unlike other FCHV tasks performed on a part-time basis, these campaigns required full-time efforts from morning until evening at a vaccination booth.

The duties associated with these national campaigns came with a renewal in FCHVs’ perceived legitimacy as health workers in the eyes both of the community and elite program managers. An INGO representative asserted in an interview in 2001:

*The nature and amount of FCHV work has changed. When they used to provide only health education, it was not very much appreciated by the community, but now because the FCHV are distributing Vitamin A capsules, giving polio drops to children and treating pneumonia in children, the community has started giving them more value and recognition.*

Community perceptions were that FCHVs were “most effective” when working with activities such as Vitamin A and polio campaigns [35].

A senior health official from Kathmandu remarked in 2001 that FCHVs were very effective in these roles, and also noted that such short-term and concrete tasks also involve no element of judgement, such as a need for a diagnosis. This comment reflects both the perceived success of FCHVs in these roles and high-level officials’ perceptions of FCHV limitations. By 2000, the NVAP was described to have become “a potent force reifying the FCHV Program,” and one that has even “raised the stature of the MOHP” [57].

The overall success of these national programs can be attributed to the broad system-level support FCHVs enjoyed for these tasks. First, their training programs were tailored to their needs. For example, the NVAP training included the use of pictorial materials and participatory, hands-on activities. Pay was also available for these activities. FCHVs received training allowances of around 75 NPR, and work stipends in some times and regions of around 100-200 NPR per day [35, 57].

### Supervision in campaign mode

Supervision for FCHVs’ routine tasks had long been spotty. A draft version of the 1992 study report stated that “supervision exists only on paper.” It continued:

*To most CHVs, supervision is a total myth… No one can express what does supervision entail …, for in the real sense, they never had one good supervision by the Health Post staff except meeting someone in the trail and asking about their work and what they are doing.*

The final report omitted this passage but did comment upon the inadequacy of existing supervision. The report related this to insufficient funding allocated to supervision, stating that “health post staff argue that field visits (supervision) are not possible with the *per diem* of 35 NPR/day provided” [34].

One further dynamic that had shifted over the years, contributing to supervision gaps, was the fact that the role of VHWs had changed during the 1990s from making house visits to largely staying in health posts.[35] The 2001 study report listed further supervision challenges for routine FCHV activities: non-payment of allowances to supervisors; geographic distance to health facility, with FCHVs located closer to facilities receiving more supervision; lack of clarity in FCHV’s job description; and health workers tasked with FCHV supervision not always receiving supervision themselves [35].

However, vitamin A and polio campaigns were the notable exceptions, where supervision was regularly provided and comprehensive. In addition to Health Post staff, doctors, supervisors, and even foreigners supervised campaign activities. This could be impersonal; FCHVs said in interviews that they did not always know who the external supervisors (a combination of World Health Organization [WHO], United Nations Children’s Fund [UNICEF], and MOHP staff) were.

An FCHV nonetheless attributed positive feelings to the tight supervision in 2012, noting that “I felt motivated when they came. If they do not come, then I feel insecure.” However, other FCHVs had differing opinions regarding their *need* to be supervised for volunteer work. One FCHV from Makwanpur noted in a 2001 interview: “Why should I be supervised when I do not get paid? I will work on my own will.”

### The increasing opportunity costs of volunteering in times of civil unrest

By 2001, FCHVs were starting to voice dissatisfaction with low incentives. Fieldnotes taken in Okhladunga district in 2001 stated that FCHVs had not received the payments they were promised for National Immunization Days, and that FCHVs were overall dissatisfied with payment rates. These allowances were generally less than the cost of hiring a day laborer to replace FCHV labor at home, [35] meaning that a day working on a polio or Vitamin A campaign could be a net loss.

Routine activities, too, could be losses for FCHVs. FCHVs also spent their own money on snacks and tea during Mothers' Group meetings,[58] and travel expenses to accompany pregnant women at institutional deliveries.

To compensate for the lack of FCHV pay, some FCHVs reported in 2001 that they also worked for NGOs, which sometimes provided relatively generous remuneration. Many of the FCHVs taking on additional NGO work were “more educated, active and free from household chores than other women in the community” [35].

All of these discussions were shot through with gendered expectations, both gendered expectations by policymakers that women be selfless and giving, and gendered expectations at the local level about who deserved pay. In a small experimental study conducted in the Pokhara district during the early years of the FCHV program, which included both men and women as ‘urban’ FCHVs, men were more dissatisfied with their incentives, demanded more incentives, reported having less time for volunteering activities, and dropped out of the program more often, compared to women [59].

Yet these gendered expectations at the local level were undergoing dramatic change. The 1990s and 2000s were a time of deep social and demographic shifts in Nepal. Most notably, the Vitamin A and polio programs were rolled out during a civil war spanning from 1996 to 2006. The war was driven by Maoist groups accusing government elites of corruption and ignoring the needs of the rural poor. The war killed 13,000 people, with thousands more disappearing or becoming displaced.

Although not much is published about how the civil war affected FCHVs themselves – nor was any substantial discussion found in our archive materials – one account states that FCHV-led national campaigns including Vitamin A, polio vaccine and family planning camps, were “hardly affected,” with Maoists at times upholding access to children’s vaccines, Vitamin A and deworming drugs [60]. Overall, it has been noted that the health care sector was among those less affected during the war [33, 60–62], although it remains the case that the war did severely affect some health workers and health posts [62, 63].

### Shifting gender roles, increasing visibility and workloads

The war did have lasting impacts on gender roles, dovetailing with broader societal shifts. In part due to male death and outmigration during the civil war years [64], women entered public space outside the home in growing numbers. This necessitated women taking a larger role in decision-making [62], and often assuming the duties of their out-migrated husbands, [65] including heavy agricultural workloads that took a substantial amount of time.

This represented a significant shift in gender norms, as women’s mobility outside the home became increasingly accepted [45]. This has been described as a “major shift from the ‘male breadwinner’ model toward an ‘adult worker model family’ (where) women add paid work to existing responsibilities for care” [66].

Yet, as women’s work responsibilities increased, FCHV work increased as well. In part because of the success of the NVAP, several additional tasks were added to the FCHVs’ duties (see Fig. 1).

FCHVs were also taking on increasing leadership responsibilities. During the early years, the 1992 study had found that FCHVs were not always able to articulate what they had learned around hygiene and disease, and that they often came across as “timid, shy, unconfident” [34].

Over the years, the Vitamin A and polio programs, as well as literacy programs, built FCHV’s confidence [57, 67–69]. Over time, too, they contributed to local planning meetings, and interacted repeatedly with health workers and other elites.[35] FCHVs were also provided opportunities to contribute to local planning as part of their monthly reporting meetings at Health Posts. They received allowances for this work; in the 2001 study, these amounted to 75 NPR and were paid by the Village Development Committee (VDC), a local government body [35].

Over time, FCHVs’ increasing role in local leadership was reflected in their being elected into local government [69]. In the 2001 study, several FCHVs had been elected as Ward and VDC Members [35].

With the increased responsibilities associated with being an FCHV, the opportunity cost of volunteering as an FCHV increased. As early as 2001, lack of time was framed as a “major constraint” and a “disadvantage” to the job by several FCHVs.

However, not all FCHVs expressed feeling overburdened by the work. One FCHV elaborated in 2011:

*Although I have many responsibilities, I manage my time between my work at home as well as at the vaccination booth. I do not feel this work is a burden. I have agreed to do the government’s work, so I should do it without feeling overburdened*.

For some FCHVs, the unpaid nature of the work created a sense of freedom in terms of time devotion. Interview notes with an FCHV from Gokulpur from 2001 stated that “she feels that when there is no money involved then there is no restriction of time.” In 2011, an FCHV stated that she got involved because she believed that “service is religion,” adding that if she really wanted money, she would have chosen other work.

FCHVs enjoyed real increased prestige coming from the expanded scope of work. Still, competing time pressures could cause family tension. FCHVs in Kanchanpur noted in 2001 that when demands for FCHV duties conflicted with work in the home, their husbands were sometimes unsupportive, angry, and even threatening. The husband of one of these FCHVs commented: “it becomes quite difficult to work without any payment as they have lots of work pending in their home. Some leave as their husbands are not happy with their work.”

Policymakers recognized the tension between growing work demands and a lack of pay. Senior officials noted in 2001 that “volunteers should not be expected to work all the time” [35]. An NGO representative said in 2001 that while FCHV work pressure had increased, the benefits had remained the same. In the same year, a District Health Officer from Okhaldhunga district said that “if MOHP can do nothing for them, they should not expect more work from FCHVs.”

### “Development-Related psychological disease”

A key dynamic behind the workload and remuneration of FCHVs was the relationship between the Nepali government and international donors. The bulk of FCHV funding was from USAID and UNICEF, with smaller amounts from UNFPA [54] as well as British, Australian and Canadian agencies, [16, 57] often channeled through INGOs and NGOs.

By the early 2000s, the FCHV program was largely viewed as a success at the national level and among donors. In 2001, a Ministry of Health report lauded the FCHVs:

*The role of the FCHVs in uplifting the health status of the community, decreasing maternal and child mortality rate, and increased use of family planning methods is commendable. … A positive attitude towards family planning has developed and community awareness on diarrhea and the importance of a clean environment has increased* [70].

By 2006, Nepal had halved its pregnancy-related mortality rate relative to 1996 [71]. FCHVs had received international acclaim for their contributions in helping Nepal reach Millennium Development Goals for child and maternal mortality [10].

This success meant that donors wanted to be involved—particularly if they could use FCHVs to achieve their own programmatic goals. A bilateral official commented, “they often would try to top-up stipends for training and provide special incentives in certain districts so that the FCHVs would do *their* [the NGOs’] activities over their regular responsibilities.”

In interviews from 2001 through to 2017, national level officials recognized that a quality FCHV program would be expensive and depended on consistent financing and active involvement from external donors. But money was not consistently available, either from international donors or the national government. During a 2001 interview, an official in Dhading district noted that while “every national and international organization wants to work with the FCHV network… none wish to allocate funds.” In a 2017 interview, a bilateral agency representative stated that a range of bilateral funders had reduced their funding support for FCHVs “because the government wasn’t kicking in as much money as the donors wanted.”

Underlying these comments was a donor unease in footing the bill for recurring costs such as regular pay. Government actors in turn expressed during interviews that they were particularly wary of accepting short-term support from donors towards FCHV remuneration, as they did not trust that such support would continue. A government report from 2001 noted that “continuity of support from donor organizations” is “one of the major constraints” of the FCHV program [70].

Both government and international actors justified their wariness to paying FCHVs by reference to their voluntarism. A district-level government official in Kanchanpur opined in 2001, “payment and voluntarism in principal are terms that do not go together.” A representative of a bilateral agency noted in 2001 that: “To emphasize the importance of voluntary action is critical and meaningful to avoid this newly spreading ‘development-related psychological disease’ in Nepal.” They continued:

*It takes quite a long time for external development workers to encourage volunteerism in rural Nepal. When they go to villages, the villagers expect development gift to be given to them freely from above. If the development workers try to change such attitudes, it takes long, long time. But as the project period is usually limited and the development workers have to show visible results within a limited time not for the target people but rather for keeping their positions or gaining their own promotion, they tend to take an easy option: money. If they give money for the target community people, they definitely become cooperative and achieve the project goal easily and with community participation.*

In this narrative, payment was an “easy out”, used as an instrumental carrot aimed at short-term gains, rather than fair compensation for the labor of the rural poor. Yet such narratives also sidestepped the real economic needs of FCHVs and ignored the actual opportunity costs of volunteering.

### Government policies to motivate FCHVs in lieu of payment

**Fig. 2:**
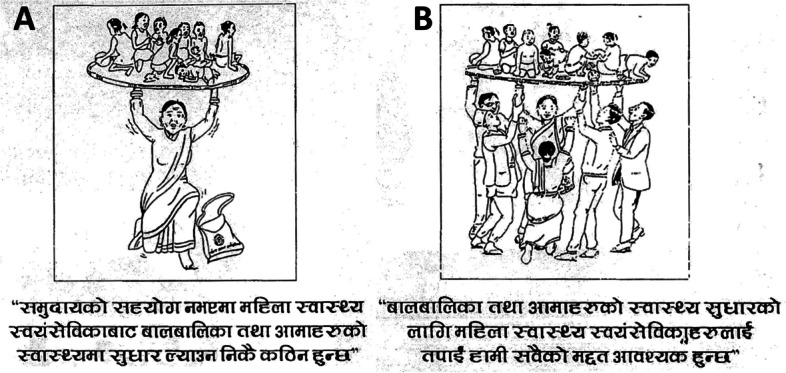
Images from a 2001 government guideline for the FCHV Endowment Fund. Notes: Image caption translations: **A** “Women health volunteers need your help to improve the health of children and mothers”; **B** “Without the support of the community, it is very difficult to improve the health of children and mothers through women health volunteers”. Source: Family Health Division, Ministry of Health [70]

In the absence of regular payment, the government introduced several policies during the 2000s to recognize and motivate FCHVs. Starting in 2001, the government encouraged local governments at the municipality/VDC level to establish an Endowment Fund to “ensure continuity of health programs at the community level” and “motivate the FCHVs” (Fig. 2) [70].

Initially, this Fund worked as capital deposited by the local municipality into an interest-bearing account. The capital itself was to remain untouched, but the interest generated could be used towards activities “for the benefit and encouragement of the FCHVs”, [70] such as tea and snacks for Mothers' Group meetings or borrowing money for community use [72]. Use towards “monthly remuneration and contingencies,” however, was strictly forbidden [70]. At first, many FCHVs were unaware of the existence of the Fund, but by 2014, sixty percent reported having used the Fund in the past year, typically for loans for educating children, domestic work or farming [58].

FCHVs also received a sari, or a dress allowance to pay for a sari, as part of their incentive package. The 2001 study found that FCHVs who had received a sari reported that the uniform raised their morale [35]. In 2010, the national government designed a standardized uniform for FCHVs: a sky-blue sari with the FCHV logo at its border [73]. Yet, receipt of saris and incentives has not always been consistent over place and time; in the 2014 national survey, only 7% of FCHVs reported having received a sari, even though the dress allowance was described as the “most important incentive in monetary terms” at the time, at approximately 4000 NPR [58].

In addition, a transportation allowance was made available for training and campaign days, and some local governments distributed in-kind incentives such as bicycles [74].

Other methods of recognition also increased over the years. FHCVs had long had opportunities to be trained as a Maternal and Child Health Worker (MCHW), a full-time, salaried, facility-based government position available to women with grade 10 schooling. Estimates from 1994 suggested that roughly one-quarter of MCHWs were formerly FCHVs [57]. By the early 2000s, FCHVs were designated as eligible for some free health care at primary health centers and district hospitals. In addition, FCHVs were given identification badges, and an annual FCHV Recognition (Appreciation) Day was established in 2003. Since 2010, it has been celebrated on December 5^th^, on the International Day of Volunteers [73, 74].

### Shifts in education and expectation

During these years, major strides were made in women’s education in Nepal, with female literacy rates increasing from 25% to 57% between 1991 and 2011. The literacy rate among FCHVs increased from 62% in 2006 to 83% in 2014 [58]. As noted earlier, this was accompanied by women in Nepal becoming more engaged in public life and as paid workers.

These shifts set up a contrast between older FCHVs, many of whom were not literate, and younger, more highly educated FCHVs. By 2010, stakeholders felt that “old age was … a challenge to adequate service delivery” among FCHVs. A national survey of FCHVs conducted in 2014 found that older FCHVs had trouble recording and reporting health data [58].

The government introduced a retirement policy in the late 2000s for FCHVs aged 60 to receive a one-time payment of 10,000 NPR for their service. Although this policy was in part intended to eliminate older FCHVs from the workforce, it was not fully successful. The 2014 national FCHV survey interviews with district stakeholders and health workers “revealed a view that the amount awarded to FCHVs at retirement should be increased to encourage older FCHVs to retire when they can no longer perform their duties” [58].

In parallel, the expectations of communities had shifted, with increasing demands for more medicalized, professional care. An INGO representative explained in a 2017 interview: “community perceptions have changed a ton. People want more care—and the health system isn’t quite providing that quality of care if you just train the FCHVs.”

By the mid-2010s, several stakeholders were skeptical regarding the ability of FCHVs to meet these changing demands. The 2014 FCHV survey notes that “some district-level respondents commented that certain health problems within the community require a qualified health professional, rather than an FCHV” [58]. The implication of this statement is that FCHVs were neither viewed as “qualified” nor “professionals.” But despite, or perhaps because of, these shifts in community, funder and program planner attitudes, FCHVs were increasingly regarding themselves as ‘workers,’ rather than volunteers.

### The rise of FCHV labor movements

By the early 2010’s, approximately 50,000 FCHVs were active across Nepal. The 2014 national survey of FCHVs showed that the majority (61 percent) of FCHVs were involved in community groups or committees such as social security committees, ward civil forums, saving and credit cooperatives, women's development committees and agricultural groups [58].

Around this time, FCHVs began expressing their dissatisfaction around insufficient pay publicly, through formalized protests in Kathmandu, and by forming labor unions to represent their rights [75]. These movements culminated into a charter of demands calling for increased allowances and additional benefits [76].

By 2018, the two main labor unions for FCHVs reported their membership bases as 12,000 and 5,000 individuals, respectively, [75] potentially representing between one-fifth and one-third of all FCHVs in Nepal. Unions raised the question of why the government’s minimum wage legislation, introduced in 2007, was not being applied to FCHVs. At that time, the minimum wage was set at 3,300 NPR per month [77].

By 2014, the government had doubled the daily allowance for training and campaign days to 400 NPR [76]. Similarly, by 2018, the dress allowance had been increased to 7000 NPR [75]. Unions took credit for these increases.

However, the government’s response to labor organizing was lukewarm at best. A Kathmandu Post article quoted a ministry official: “Since this is voluntary work, the Ministry cannot allocate a salary for such a large number of volunteers” [78].

### Questions of legitimacy and futures for FCHVs

Unionizing had the unfortunate effect of deepening some government officials’ skepticism about the FCHV program. A representative of a bilateral agency summarized the government position in 2017: “Because we cannot offer salary to 50,000 plus FCHVs, so why not phase out this program.”

Another high-level official in Kathmandu explained:

*One of the reasons [that the government is trying to get rid of FCHVs] is they [FCHVs] mobilized a few years ago. They had all kinds of demands. Political leaders got irked. They put pressure on the bureaucrats. So, the bureaucrats said, “let’s just get rid of them.”*

This push to eliminate the FCHV program, yet another INGO representative based in Kathmandu explained, came in part from a feeling that FCHVs did not have a place in the vision of a medicalized health system for Nepal.

In 2017, an official indicated in an interview that the government wished to limit the role of FCHVs to the sharing of health information and mass campaigns, leaving other activities to staff with professional degrees. Although FCHVs were initially tasked with treating childhood pneumonia cases with antibiotics as part of the 2008 Community-Based Newborn Care Package, [79] by 2014, government policy had rendered FCHVs ineligible to do so [80, 81]. The 2015-16 Department of Health Services Annual Report stated that FCHVs’ role had been “redefined and limited” to “health promoters/counsellors rather than health service providers,” with tasks such as counselling, dispensing essential commodities and referral of newborns and children exhibiting warning signs to higher levels of care [82].

An INGO representative commented in 2017 that there were two “schools of thought” about FCHVs. On the one hand, they said, INGOs were “pushing forward the FCHVs—telling the government not to underestimate their power.” On the other hand:

*The government is looking at other alternatives, because of the increasing demand by FCHVs for incentives, etc. And, by people for comprehensive health services—can the FCHVs really meet those demands? …. Do we really need FCHVs as educators when educational status and access to media through phones is so much higher than it used to be?*

These thoughts of eliminating FCHVs came amidst a major shift in the governance of the program: the decentralization of the responsibility for primary health care and public health from the central government to the recently-formed municipalities, or *palikas*. This structure of federalization was formalized in the 2015 constitution. This has allowed municipalities the option to keep existing FCHVs and increase their incentives or introduce alternative cadres. The latter has already happened in some areas such as Dolakha and Achham districts where paid CHWs have been introduced alongside FCHVs [83, 84].

However, given the breadth and success of the FCHV program, along with the political power held by the 50,000+ workforce, eliminating the cadre is not a simple proposition. Respondents at national and local levels said in 2014 and 2017 interviews that it was difficult to get FCHVs to retire [58]. This speaks to the dedication of FCHVs towards their role, as well as the strong identity they have built over their decades-long service. Yet, the future and legitimacy of these workers in the face of shifting community demands, remains uncertain.

## Discussion

Looking across four decades of the FCHV program’s history illuminates some themes that resonate across time and also with current experiences.

FCHVs themselves gained much of value from the work. It gave them access to trainings and privileged medical knowledge and resources [5], and along with them, they have earned community respect and legitimacy as ‘health workers’. Over time, they took on positions of leadership, including winning local elections. Our work provides further evidence on the importance of social recognition, collateral leadership opportunities and religious merit as motivational factors for FCHVs [9, 74, 85].

Somewhat paradoxically, single-disease, donor-funded vertical programs were critical contributors to this legitimacy, as shown in our work and also noted by other scholars [86, 87]. Although the Integrated Community Health Program was a move towards a primary care-approach with broad-based health workers, vertical programs like the NVAP, and the vertical planning and management of the FCHV program, are evidence that much health programming has been fragmented and donor-driven [80, 88, 89]. Yet FCHVs’ engagement with these vertical programs—and the funding and high-quality trainings that these vertical programs provided—often benefited their broader legitimacy.

Another trend throughout time is that FCHVs have gained legitimacy through providing tangible commodities and curative treatments. Where utilization and awareness of FCHV services remains low, one contributing factor is FCHVs lacking medicines [90]. The potential removal of some curative services from FCHVs’ roster [86] could threaten overall FCHV legitimacy. It remains to be seen whether the engagement of FCHVs in non-communicable diseases, [91–94] which now contribute to the majority of deaths in Nepal, [95] will shift community expectations and views of FCHVs. While it has been argued that CHWs risk becoming obsolete in the eyes of policymakers "once more sophisticated health services are available”, [96] others claim that CHWs should continue to be essential health workers even after the burden of disease from communicable and maternal and child health conditions has reduced [97].

We also show that legitimacy has long been tied to the work context. FCHVs have faced issues of workload and pay throughout their engagement as health workers.

In the debate around CHW incentivization, it is sometimes suggested that external incentives such as pay may reduce intrinsic motivation to serve and help one’s community members, [20, 29, 98, 99] based on the *motivation crowding theory* [100]. For Nepal’s FCHVs, it has been argued that their volunteer status is exactly what has earned them their community respect and trust [19] as payment would otherwise threaten their social status in their communities [101].

But this ignores the deeply gendered aspects of CHW labor and remuneration [23, 28, 102–105].

Nepal’s early experiments with male CHW cadres parallels experiences in India and Ethiopia, which also originally had male CHW cadres that were abandoned for all-female cadres. In all three countries, these shifts occurred in part because of a desire to deliver maternal and child health care in a culturally appropriate way. But these shifts were also about gendered expectations around labor compensation [24, 103]. In India, the government phased out male CHW cadres when they started protesting to demand higher payment [24, 106–108]. The shifts from male to female volunteer CHW workforces are one demonstration of women being expected to “do more for less” compared to men [23]. In all of these contexts, work shows that CHWs are not economically empowered by their work done for health systems [103, 109–111]. Our findings regarding the opportunity costs of volunteering also shed light on the myth on women having “spare time” to volunteer [112].

This is underlined by the widespread documentation of the dissatisfaction among FCHVs towards their small incentives – as noted in our study, as well as in several other studies [9–11, 101]. Our material supports the notion that the dissatisfaction among FCHVs is related to the increased opportunity costs of volunteering, i.e. the replacement costs of a day laborer being higher than the incentives that FCHVs receive for their work [9].

The remuneration debate deserves further attention at this particular moment. Very recently, UN agencies and international organizations have started expressing concern over the uncompensated and gendered nature of CHW work [105, 113, 114]. A 2017 International Labor Organization (ILO) report stated that FCHVs are an example “of a system built on women’s unpaid or low paid and devalued work” [115]. Even UNICEF – one of the most important funders of the FCHV program from the outset – stated in a 2022 report that Nepal’s health system reliance on unpaid FCHVs “appears to reinforce gender disparities… the possibility remains that women are being exploited and are unable to gain economic independence” [116].

Our findings also highlight how sociopolitical events, such as major transitions in governmental regimes, necessarily affect the ‘accountability ecosystem’ surrounding CHWs.[117, 118] The transition from the Panchayat regime to parliamentary democracy had impacts for the removal of FCHV monthly stipends as well as their selection processes. Since Nepal is today characterized as a ‘fragile’ and ‘post-conflict state’ [119] given the relative recency of the civil war, we urge greater attention to sociopolitical factors that continue to shape the lives of FCHVs.

Preliminary evidence on Nepal’s current shift to a federal republic, which decentralized the responsibility of community health to local governments (*palikas*), suggests that the division of labor and authority between *palikas*, provinces and the central government remains unclear [120, 121]. The federal government may still at times retain control of policy, planning, and financing [119, 120, 122]. On the other hand, some observers have claimed that federalization has led to better provision of home-based health services to vulnerable populations, given that local governments have “health as their top priority” [123]. While our archived material did not touch upon the impacts of federalization on FCHVs, federalization offers *palikas* the opportunity to either strengthen the existing FCHV program or replace them with alternative cadres. Some local governments have already been experimenting with the latter through public-private-partnerships, such as the implementation of paid, professionalized and fully-supported CHWs in Achham and Dolakha districts [84]. These models are explicitly grounded in the WHO recommendations on engaging CHWs, [124] and notably, provide CHWs a salary that is higher than the national minimum wage [83].

This article also provides a contribution to the literature by tracing available information on FCHV labor movements, adding to the scant literature on the topic globally, [24–28, 107, 118, 125–127] and particularly for Nepal’s FCHVs [29]. Increasing protest activity and unionization over time could be argued to be a hallmark of FCHVs developing a sense of their own professionalization as *workers*, rather than *volunteers*. Yet, legitimate challenges to this vision remain. Schaaf and colleagues argued that collective voice or action may be especially difficult for CHWs operating in hierarchical government health systems, given that CHWs are at the lowest tier of the hierarchy [118]. In Nepal, we show how government officials hesitate to regularize FCHVs as 'employees' partly because of the long-term unreliability of donor funds. At the core of the FCHV remuneration debate, thus, lies a delicate government-donor dynamic which is likely to be impacted by the demands brought by federalization [16, 116]. The remuneration debate thus remains unsolved, but history suggests that it will be with us as long as FCHVs are asked to provide labor without adequate pay.

Our other findings are in line with previous research surrounding the importance of institutional support for the well-being and motivation of FCHVs, aside from remuneration. WHO guidelines emphasize that CHW compensation should be commensurate with workload [124]. Our work illustrates the increase in FCHV workload over time, echoing existing literature [10, 19, 80]. There is also widespread guidance and evidence on the importance of supportive supervision and adequate supplies for CHWs, [124, 128–132] both of which have been longstanding challenges for Nepal’s FCHVs [85, 133].

Our study has some limitations. First, we relied heavily on primary materials collected by one person, and from three research studies shaped heavily by that same person. The materials are thus geographically bound to their respective study locations. Further, the body of work we reviewed is heavily influenced by Dr. Justice’s interests, and their anthropological sensibility. This is, of course, in some ways a bias. At the same time, Dr. Justice was an experienced and comprehensive collator and chronicler of FCHV information over many years, resulting in a collection of FCHV-related materials over the years that to our knowledge is more comprehensive than any other collection in existence. We tried to our best ability to fill gaps in our understanding and address questions that arose from our materials, by delving into published grey and peer-reviewed literature where questions arose.

Given that our focus was materials in this archive, our work does not cover recent important events affecting FCHVs. The archive contained materials overlapping only with the first few years of federalization and did not contain materials pertaining to the COVID-19 pandemic.

In addition, the quality of the data we had to work with was sometimes patchy. Some of the researchers engaged in data collection in both the 1992 and 2001 studies lacked experience with qualitative data collection, something which manifested in data that was often thin. Interviews were not regularly audio recorded, and hand-written notes were often pithy. Where recordings did exist, we often found that time had corroded the tapes.

Despite these limitations, we found great value in working with archives. We found data material in draft reports, margin notes, paper memos and faxes particularly interesting. While the reasons behind material in draft reports being frequently omitted from final reports are unclear, this highlights how research narratives contained in ‘final’ reports are filtered through the interests of ‘elite’ institutions funding such research – and the FCHV program itself.

## Conclusion

A look at the 35-year trajectory of Nepal’s female community health volunteers (FCHVs) highlights that gendered aspects of workload and pay are policy dilemmas that have persisted since the beginning of the program. FCHVs replaced early male cadres, in part due to programmatic goals around maternal and child health, but also because of gendered expectations around remuneration. This history also illustrates how health system support in the form of supervision and payment to workers can be important in rendering CHWs legitimacy as health workers. Indeed, the removal of FCHV stipends during the 1990s impacted not only FCHVs’ family dynamics, but their credibility in their communities. Our analysis also highlights the importance of gender at the level of the household, in determining whether FCHVs have time available to volunteer for health tasks. Nepal’s political history has impacted the work and life of FCHVs, both directly and indirectly. Against the background of these longstanding dynamics, the rise of labor movements over the past decade and a half highlight FCHVs’ nascent identity as workers. Historical dilemmas around pay and workload continue to color current-day policy debates around the future and relevance of FCHVs.

Across public health, an awareness of history is useful in understanding the present and avoiding past mistakes. These histories are often not well archived, and risk getting lost. But the lessons of the past have much to offer the debates of the present.

### Supplementary Information

**Supplementary Material 1**

## Data Availability

To protect the study participants’ anonymity, data from individual studies obtained through semi-structured interviews, focus group discussions and participant observation, are not publicly available. Data may be made available on reasonable request.

## Abbreviations

CHL: Community Health Leader
CHW: Community Health Worker
FCHV: Female Community Health Volunteer
INGO: International non-governmental organization
MCHW: Maternal and Child Health Worker
MOHP: Ministry of Health and Population
NGO: Non-governmental organization
NPR: Nepali rupees
NVAP: National Vitamin A Program
PHV: Panchayat Health Volunteer
UNICEF: United Nations Children's Fund
USAID: U.S. Agency for International Development
VDC: Village Development Committee
VHW: Village Health Worker
WHO: World Health Organization
