# Supplementary material for: An anthropological history of Nepal’s Female Community Health Volunteer program: gender, policy, and social change
Source: Int J Equity Health. 2024 Apr 13;23:70. doi: 10.1186/s12939-024-02177-5 (PMC11015651; doi:10.1186/s12939-024-02177-5)
Supplement: Supplementary file 1 — Supplementary Material 1 [file 12939_2024_2177_MOESM1_ESM.docx]

# Additional File 1

Table showing coding tree generated on MaxQDA, that was used to find themes of interest. Codes and their frequencies are given for higher-order (Level 1; parent) and lower-order (subcategories / child; Level 2) levels.

**Supplementary Table 1. MaxQDA Coding Tree showing Frequencies for Level 1 (parent) and Level 2 (child) codes.**

| **Level 1** | **Level 2** | **Frequency** |
| --- | --- | --- |
| **Health systems** | | **749** |
|  | History of FCHVs | 54 |
|  | Leadership & Planning | 49 |
|  | Financing | 44 |
|  | Cooperation & Coordination | 45 |
|  | Government | 166 |
|  | NGOs | 133 |
|  | Health system | 132 |
|  | INGOs | 125 |
| **Handwritten scribbles (margins)** | | **175** |
| **Auxiliary health workers (other)** | | **181** |
|  | FCHV Precursors | 66 |
| **Performance** | | **493** |
|  | Community perceptions & relations | 319 |
| **Exploitation** | | **12** |
| **Training** | | **207** |
| **Gender** | | **96** |
|  | Female empowerment | 10 |
|  | Gender - Male | 43 |
| **Cultural context** | | **49** |
| **Work Challenges** | | **200** |
| **Advocacy for FCVHs** | | **23** |
| **Selection** | | **310** |
|  | Tenure | 60 |
| **Areas of Responsibility** | | **1025** |
|  | Patient fees and OOP | 29 |
|  | Medicines, med. devices, technologies | 122 |
|  | Absorptive capacity | 53 |
|  | Social groups | 33 |
|  | Geographic differences | 57 |
|  | Polio | 88 |
|  | Vit A | 88 |
|  | RMNCH incl. Mothers Groups | 277 |
|  | Immunization | 14 |
| **Work life balance** | | **77** |
|  | Multiple jobs | 33 |
| **Benefits** | | **68** |
|  | Volunteerism | 20 |
|  | Training allowances | 46 |
|  | Renumeration | 205 |
| **Motivation** | | **68** |
| **Working conditions** | | **232** |
|  | Environmental conditions | 17 |
|  | Equipment, Supplies | 61 |
|  | Supervision | 140 |
|  | Teamwork | 11 |
| **Family of FCHV** | | **33** |
| **"Development disease"** | | **34** |
| **Socio-political context** | | **50** |
| **Sustainability/Long-term** | | **21** |
| **Evaluation & Research** | | **19** |

NOTE: Text segments could be coded at Level 1 or Level 2. Thus, the sum of the individual unbolded figures at Level 2 do not necessarily sum up to the Level 1 figure.
